# Transmission Potential of Influenza A(H7N9) Virus, China, 2013–2014

**DOI:** 10.3201/eid2105.141137

**Published:** 2015-05

**Authors:** Adam J. Kucharski, Harriet L. Mills, Christl A. Donnelly, Steven Riley

**Affiliations:** London School of Hygiene and Tropical Medicine, London, UK (A.J. Kucharski);; National Institutes of Health, Bethesda, Maryland, USA (A.J. Kucharski);; Imperial College London, London (A.J. Kucharski, H.L. Mills, C.A. Donnelly, S. Riley)

**Keywords:** H7N9 virus, influenza, infectious disease reservoir, viruses, China

## Abstract

To determine transmission potential of influenza A(H7N9) virus, we used symptom onset data to compare 2 waves of infection in China during 2013–2014. We found evidence of increased transmission potential in the second wave and showed that live bird market closure was significantly less effective in Guangdong than in other regions.

From February 19, 2013, through April 22, 2014, a total of 429 cases of influenza A(H7N9) virus infection in humans in China were reported and occurred in 2 outbreak waves. During the first wave in spring 2013, live bird markets were closed in several parts of China (*1*,*2*); these market closures substantially reduced the risk for infection in affected regions (*3*). During a second wave in autumn 2013 (*4*), markets were again closed in some provinces (*5*–*7*). Analysis of the largest clusters of subtype H7N9 virus infection in 2013 suggested that the basic reproduction number (R_0_, the average number of secondary cases generated by a typical infectious host in a fully susceptible population) was higher in some clusters than in others (*8*,*9*), although the absence of sustained transmission implied that R_0_ was less than the critical value of 1. To determine the transmission potential of influenza A(H7N9) virus in the first and second waves in 2013, we compared symptom onset data. We also measured the extent to which market closures in 2014 reduced spillover hazard (i.e., risk for animal-to-human infection). 

## The Study

We focused on the locations of the 6 largest outbreaks: Shanghai, Zhejiang, and Jiangsu (first wave) and Guangdong, Zhejiang, and Jiangsu (second wave). To infer market hazard and human-to-human transmission potential, we used a statistical model of infection spillover (*9*). We assumed that cases could be generated in 1 of 2 ways: on each day, the expected number of reported cases was equal to the sum of animal exposure and secondary cases generated by earlier infectious hosts (Technical Appendix). Use of such a framework enables estimation of the degree of human-to-human transmission from symptom onset data and of exposure hazard from markets; the accuracy of these estimates is greatly improved when the timing of a sudden change in hazard, such as a market closure, is known (*9*). We therefore constrained the timing of the drop in exposure hazard to reported market closure dates (Technical Appendix Table 1). We also estimated R_0_ for each of the 6 outbreaks. For patients with known exposure, cluster reports suggest that the serial interval (time delay between symptom onset in primary and secondary case-patients) could be 7–8 days (Technical Appendix Table 2). We therefore assumed a serial interval of 7 days for our main analysis and tested a range of values from 3 to 9 days during sensitivity analysis. We adjusted for potential delays between symptom onset and case report on the basis of the distribution of delays to date (Technical Appendix Figure 1).

**Figure 1:**
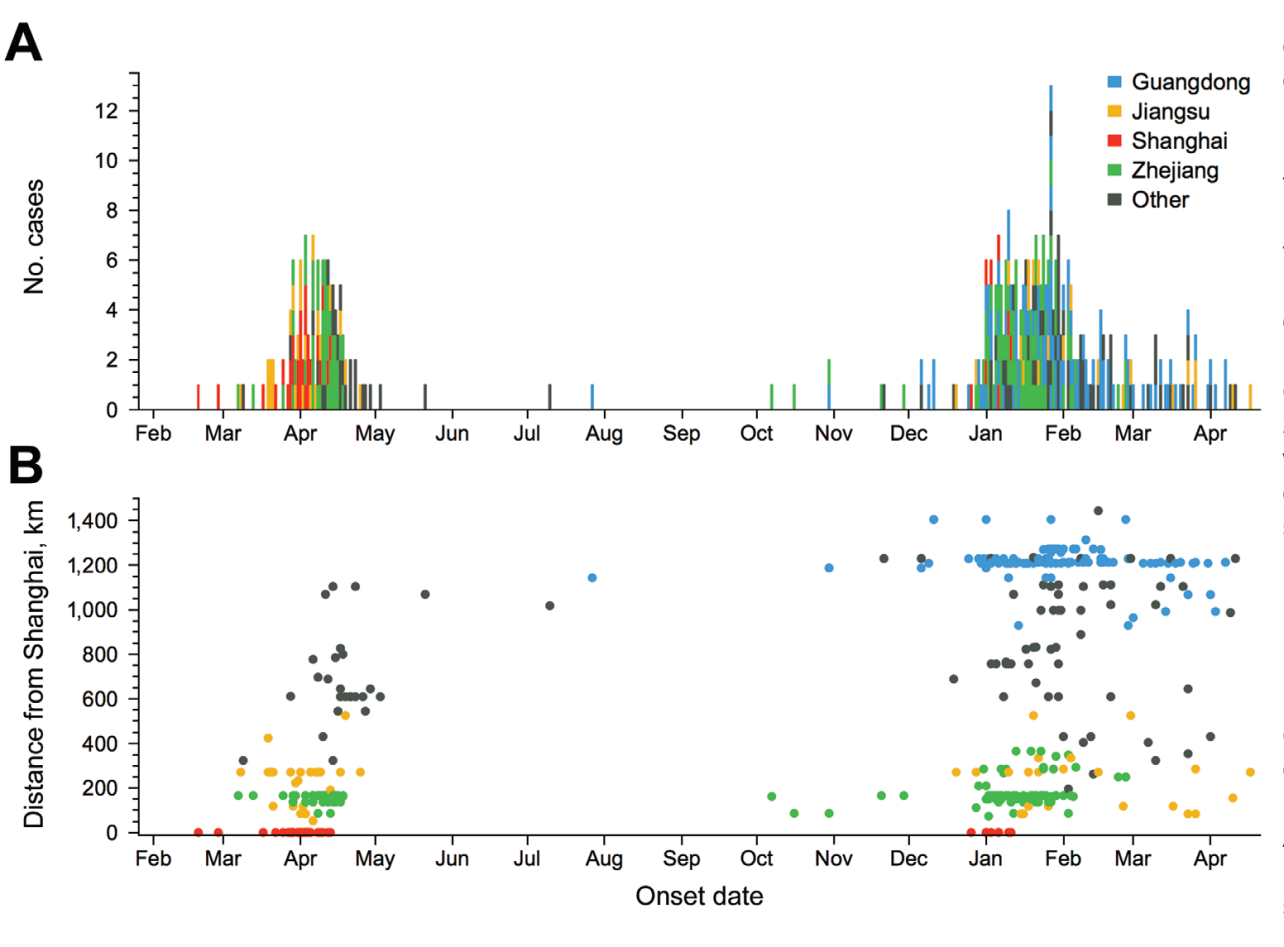
Spatial and temporal distribution of reported cases of influenza A(H7N9) virus infection among humans, China, 2013–2014. Onset of the first case in wave 1 was February 19, 2013 (although the case was not reported until the end of March 2013); onset of the last case in wave 1 was July 27, 2013; only 4 cases occurred in May–July 2013. Onset of the first case in wave 2 was October 7; onset of the last case in our time series was April 17, 2014. A) Case onset reports across all regions. Colors indicate the 4 largest geographic clusters; black indicates all other cases. B) Spatial pattern of reported cases. Points show geodesic distance between the first reported case of influenza A(H7N9) virus infection (in Shanghai) and location of each subsequent reported case. Cases are colored by region as in panel A.

During the first wave, cases were initially concentrated around Shanghai; reports centered on the city and neighboring Zhejiang and Jiangsu (Figure 1, panel A). A wave-like relationship between location and onset timing was apparent; distance between the location of the first case-patient in Shanghai and subsequent case-patients increased over time (Figure 1, panel B). The pattern of cases at the start of the second wave suggests that infection did not spread outward from a single source; in October 2013, initial cases occurred in Guangdong and Zhejiang.

**Figure 2:**
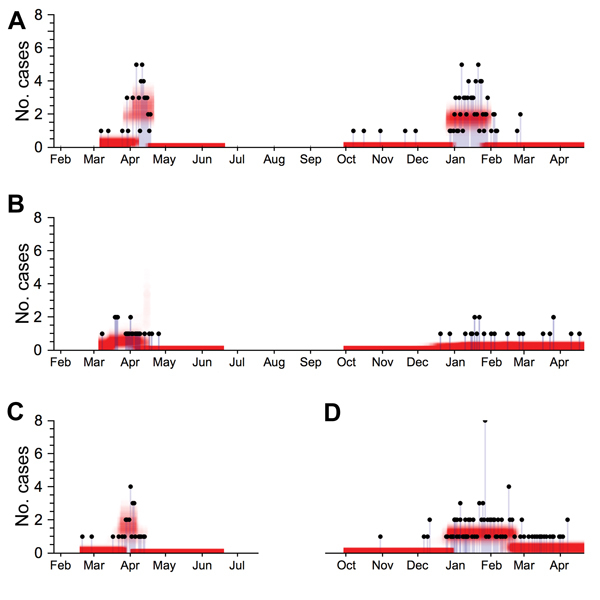
A posteriori probability estimates of spillover hazard for influenza A(H7N9) virus infection in China, by region. Black dots show total number of reported influenza A(H7N9) virus cases for which symptom onset occurred on a given date. Red shading shows a posteriori probability estimate of spillover hazard (i.e., the expected number of cases resulting from animal-to-human transmission on each day). A serial interval of 7 days was assumed. A) Zhejiang, 2013–2014; B) Jiangsu, 2013–2014; C) Shanghai, first outbreak wave, 2013; D) Guangdong, second outbreak wave, 2013–2014.

We used our statistical model to estimate the relative contributions of animal-to-human and human-to-human transmission. In Zhejiang, Shanghai, and Guangdong, market hazard clearly increased and decreased at the start and end of the outbreak, respectively (Figure 2). We also estimated R_0_ for different regions over the 2 outbreak waves (Table). Although our estimates for Jiangsu did not change significantly between the 2 waves, for Zhejiang, R_0_ was significantly higher for the second wave than for the first wave in spring 2013 (p = 0.045). We estimated R_0_ to be 0.06 (95% credible interval [CrI] 0.00–0.25) in the first wave and 0.35 (95% CrI 0.15–0.65) in the second.

**Table:** Estimates of human-to-human transmission and effectiveness of live bird market closures, China, 2013–2014*

Region, outbreak wave	Total no. cases	R_0_ (95% CrI)	Human-to-human transmission, no. cases (95% CrI)	Hazard reduction, % (95% CrI)
Shanghai, first	29	0.32 (0.06–0.60)	11.0 (2.3–14.8)	99 (95–100)
Jiangsu				
First	23	0.24 (0.03–0.69)	6.7 (2.0–12.2)	97 (80–100)
Second	26	0.13 (0.01–0.41)	2.9 (0.1–8.7)	NC
Zhejiang				
First	46	0.06 (0.00–0.25)	3.8 (0.8–12.4)	99 (97–100)
Second	92	0.35 (0.15–0.65)	32.5 (17.3–48.9)	97 (92–99)
Guangdong, second	103	0.16 (0.01–0.54)	16.7 (1.0–48.6)	73 (53–89)

*A serial interval of 7 days was assumed. For sensitivity analysis, see Technical Appendix. CrI, credible interval; NC, not calculated; R_0_, reproduction number (average number of secondary cases generated by a typical infectious host in a fully susceptible population).

Using our estimates for R_0_ and market hazard, we estimated the number of cases in each outbreak that resulted from human-to-human rather than animal-to-human transmission. We found evidence of a small but significant amount of transmission between humans in the first and second waves (Table). Our findings agree with reports of possible human clusters in the first wave (*1*,*10*–*12*) and corroborate media reports of possible human clusters in Zhejiang and Guangdong during 2013–2014. We identified 5 clusters during the first wave (February–April 2013) and 8 clusters during the second wave (November 2013–May 2014); the clusters in both waves had median size of 2 cases per cluster (Technical Appendix Table 2). These conclusions were robust under different assumptions about the duration of serial interval (Technical Appendix Figures 2, 3).

During the second wave, market closures in Zhejiang began on January 22, 2014, and ended on January 26, 2014 (Table). The reduction in spillover hazard after these closures was significant. We estimated that closures for a serial interval of 7 days reduced hazard by 97% (95% CrI 92%–99%). During 2013, estimated effectiveness was similar in Zhejiang (99%; 95% CrI 97%–100%) and Shanghai (99%; 95% CrI 95%–100%). These estimates are in agreement with those from other analyses for the first wave (*3*). The 95% CrI was broader for Jiangsu, however, where estimated effectiveness was 97% (95% CrI 80%–100%). In Guangdong, Guangzhou markets closed on February 16, 2014, and reopened on February 28; markets in other cities in Guangdong closed around the same time for 2 weeks. Our results suggest that these closures reduced hazard by 73% (95% CrI 53%–89%). This reduction was significantly smaller than that for Shanghai and Zhejiang (p<0.01). Our result was robust at different serial intervals of infection (Technical Appendix Figure 4).

Despite the effectiveness of closures during the first wave, interventions in most regions were delayed until after the Chinese New Year (January 31, 2014). Some regions are investigating alternative market practices: Guangzhou has implemented a trial of a permanent ban on live poultry sales in certain markets, potentially to extend over the entire city by 2024 (*5*). Our results support recommendations made after the first wave of outbreaks in 2013 (*3*), which suggest that prompt closure of markets could lead to substantially fewer infections. However, our finding that the relative effectiveness of the shorter closure in Guangdong was lower suggests that such interventions are needed for a sufficiently long time to prevent recurrence.

Our study has limitations. First, case data were insufficient for us to jointly infer serial interval and transmissibility. We therefore tested our results against a wide range of plausible assumptions about the serial interval of infection (Technical Appendix). We also assumed that the market hazard increased and decreased in a simple stepwise manner (Figure 2). Local market density could also influence the size of spillover hazard and, hence, effectiveness of interventions (*13*). If the market hazard could be better characterized (e.g., by longitudinal serologic surveillance [*14*]), the accuracy of our estimates would probably be improved (*9*). When estimating R_0_, we did not incorporate individual-level variability in transmission and potential superspreading events. However, the framework that we used can still produce reliable estimates of R_0_ when a population contains superspreaders (*9*).

## Conclusions

We found no evidence of reduced human-to-human transmission between the 2 waves. For a serial interval of 7 days, we estimated that R_0_ increased in Zhejiang. Furthermore, the effectiveness of live bird market closures varied between regions; short-term closures were substantially less effective than interventions in other regions. These results emphasize the value of prompt and sustainable control measures during outbreaks of influenza A(H7N9) virus infection.

Technical Appendix Additional data for influenza A(H7N9) virus transmission model and effectiveness of live bird market closure, China, 2013­—2014.
