# Supplementary material for: Transmission Potential of Influenza A(H7N9) Virus, China, 2013–2014
Source: Emerg Infect Dis. 2015 May;21(5):852–5. doi: 10.3201/eid2105.141137 (PMC4412215; doi:10.3201/eid2105.141137)
Supplement: Technical Appendix — Additional data for influenza A(H7N9) virus transmission model and effectiveness of live bird market closure, China, 2013­—2014. [file 14-1137-Techapp-s1.pdf]

Article DOI: http://dx.doi.org/10.3201/eid2105.141137

Transmission Potential of Influenza A(H7N9) Virus, China, 2013–2014

Technical Appendix

Data

Using WHO reports and news reports, we collated a line list of reported influenza A/H7N9 cases between 19th February 2013 and 22nd April 2014. In this period there were 429 cases in total, split into two outbreak waves: 144 cases in the spring 2013 wave, which started on 19th February 2013, and 285 cases in the 2013/2014 wave, which began on 7th October 2013 (Figure 1A).

Transmission model

In the model, human cases could be generated in one of two ways [1]. First, they could come from exposure to live bird markets (LBMs). We defined $h_A(t)$ to be the expected number of new human cases with onset on day t due to market exposure. We assumed this to be a step function with S steps and $S - 1$ change points. Cases could also come from human-to-human transmission. In our model, infected individuals had an infectiousness profile described by a Poisson distribution with mean λ, the serial interval of the disease. The number of new infections generated by each infectious individual was dependent on R_0; because there were few total infections relative to the population size, we assumed that depletion of the susceptible pool did not affect the dynamics [2]. We defined $h_H(t)$ to be the expected number of new human cases with onset on day t due to previous human cases,

$$h_H(t) = \sum_{i=1}^{I_t} R_0 \frac{\lambda^{t-d_i} e^{-\lambda}}{(t-d_i)!} \qquad (1)$$

where d_i was the time infected, hence $t - d_i$ was the time since individual i was infected, and I_t was the total number of infected individuals at time t.

We assumed that the number of new human cases on a given day, N_t, followed a

Poisson distribution with mean $h_A(t) + h_H(t)$. Hence the expected number of cases on day t was given by:

$$\mu_t = \begin{cases} h_A(t,\theta) \text{ if } t = 0; \\ \sum_{i=1}^{\min(k,t)} R_0 N_{t-i+1} \frac{\lambda^i e^{-\lambda}}{i!} + h_A(t,\theta) \text{ if } t > 0 \end{cases} \quad (2)$$

where k is the maximum value the generation time distribution can take.

We used a likelihood-based approach to estimate epidemiological parameters. For a time series of observed human onsets $\{N_t\}_{t=1}^{T}$, the likelihood of our parameter set is [3]:

$$L(\theta | N) = \prod_{t=0}^{T-1} \frac{\mu_t^{N_{t+1}} e^{-\mu_t}}{N_{t+1}!} \quad (3)$$

The expected number of cases, μ_t, depends both on the shape of the spillover hazard function, $h_A(t, \theta)$, and human-to-human transmission parameters, R_0 and λ. For five of the outbreaks, we assumed that the temporal change in market hazard followed a step-wise hazard function with three steps. The hazard function had five parameters: 3 parameters controlling the relative amplitude of spillover infections, and 2 controlling the timing of the increase and decrease in hazard. We constrained the timing of the drop based on reported market closure dates (Table S1). In the first wave, we assumed that market hazard decreased on a date within 7 days either side of 6th April 2013; in Shanghai, we assumed closure occurred on on 10th April 2013 (± 7 days) in Jiangsu and on 16th April 2013 (± 7 days) in Zhejiang. During the second wave, we assumed that hazard dropped on 26th January 2014 (± 7 days) in Zhejiang and on 16th February 2014 (± 7 days) in Guangdong. As we could not find reports of market closures in Jiangsu in 2014, we used a two-step hazard function for this outbreak, with only an increase in hazard. As well as market hazard, we estimated the basic reproduction number, R_0, for each of the six outbreaks.

For individual sets of parameter estimates, we used a fixed serial interval, λ. For patients with known exposure, the incubation period of H7N9 infection had a median of 6 days [4] and cluster reports suggest serial interval could be around 7-8 days (Table S2). In our main analysis, we therefore assumed a serial interval of 7 days. However, there is evidence that serial interval for seasonal influenza can be as low as 3-4 days [5]. During sensitivity analysis, we tested a

range of values from 3 to 9 days. We also adjusted for potential delays between symptom onset and case report based on the observed distribution of reporting delays (Figure S1). We assumed that the delay between onset and report followed a normal distribution: based on H7N9 cases reported up to 22nd April 2014, the reporting delay has a mean of 9.0 days and standard deviation of 3.3 days.

Model inference was performed using the full likelihood and Markov Chain Monte Carlo (MCMC) over the space of possible parameter values. We assumed that each parameter was positive, with a flat prior distribution.

The size distribution of human clusters can also be used to estimate the reproduction number of an infection [6]. However, estimation of R_0 from the total outbreak size distribution is implicitly conditional on the infection having so far failed to cause a large epidemic. This condition means it is not possible identify whether R_0 is greater or less than one, and hence whether it has pandemic potential [7]. Moreover, cluster size analysis does not account for change in exposure hazard over time, which can affect the accuracy of transmissibility estimates [8]. The method we here is robust to both of these issues: we did not make the implicit assumption that $R_0 < 1$, and we incorporated information on the temporal change in market hazard when estimating transmission potential.

Calibration of animal-to-human component of model

Before estimating R_0, we calibrated the market exposure component of the model without the presence of human-to-human transmission. LBMs were closed in Guangdong and Zhejiang in spring 2014. Previous work has shown that a 3 step hazard function performed best according the Bayesian Information Criterion (BIC) for the first wave [1]. We also found most support for 3 step function in 2014 (Table S3). Because we found no reports of closures in Jiangsu in 2014, we assumed a 2 step hazard function for this region.

Technical Appendix Table 1. Details of LBM closures in China in 2014*

Province	City	District	Markets closed	Markets open	Notes
Zhejiang	Hangzhou	Various	22/01/2014, 24/01/2014 and 26/01/2014	unreported	Also closed circuses with live animals
	Hangzhou	Jianggan, Xiacheng, Shangcheng, Gongshu and Xihu	15/02/2014	permanent ban expected	Permanent ban on trading of all birds, for meat and pets. Frozen poultry only to be sold from the end of February.
	Hangzhou	Xiaoshan and Yuhang	15/02/2014	15/05/2014 (expected)	Ban on trading of all birds, for meat and pets.
	Ningbo	Main city districts: Haishu, Jiangdong, Jiangbei , Yinzhou	26/01/2014	unreported	Also stopped people from flying their homing pigeons, closed zoos and scenic bird tours
	Jinhua		by 26/01/2014	unreported	
	Shaoxing		by 26/01/2014	unreported	
Anhui	Anqing	Urban area of Anqing city including Susong County	09/02/2014		
Guangdong	Guangzhou		16/02/2014	28/02/2014	
	Zhongshan		10/02/2014	23/02/2014	
	Shenzhen		31/01/2014	13/02/2014	
Shanghai	Shanghai		31/01/2014	01/05/2014	Extra measures now in place: with extra measures - now will close one day a week for cleaning / sterilization
Hong Kong	Hong Kong		29/01/2014	19/02/2014	The ban on live chicken imports from China will continue for four months. The government intends to screen imported poultry at a holding site.

* In the model, we assumed the drop in hazard resulting from LBM closure in the second wave occurred in Guangdong on 16th February 2014 (± 7 days); and in Zhejiang on 24th January 2014 (± 7 days). For the first wave, based on reported closures [1], we assumed a drop in hazard in Shanghai on 6th April 2013 (± 7 days); in Jiangsu on 23rd March 2013 (± 7 days); and Zhejiang on 31st March 2013 (± 7 days). Dates as reported in public news sources, including Shanghai Daily, Xinhuanet, Guangzhou Daily, Anhui News, China Daily.

Technical Appendix Table 2. Possible human clusters as identified from linelist data and news reports*

Cluster ID	Region	Case ID	Onset Date	Notes
1	Shanghai	1	19-Feb-2013	Father of ID 73 and 76
		73	unknown	Son of ID1
		76	unknown	Son of ID1
2	Shanghai	12	27-Mar-2013	Wife of ID45
		45	02-Apr-2013	Husband of ID12
3	Beijing	44	11-Apr-2013	Child
		88	none	Child, asymptomatic contact of ID44 (parents had bought chickens from parents of ID44)
4	Shandong	106	16-Apr-2013	Father of ID127
		127	27-Apr-2013	Child of ID106
5	Jiangsu	6	21-Mar-2013	Daughter of ID120
		120	08-Mar-2013	Father of ID6
6	Guangdong	165	03-Jan-2014	Father of ID202
		202	14-Jan-2014	Reported daughter of ID165
7	Zhejiang	212	13-Jan-2014	Suspected family cluster (father)
		229	20-Jan-2014	Suspected family cluster (daughter)
		254	23-Jan-2014	Suspected family cluster (mother)
8	Hunan	280	24-Jan-2014	Father of ID286
		286	30-Jan-2014	Daughter of ID280
9	Guangxi	300	27-Jan-2014	Mother of ID310. Traveller, developed fever in Guangdong
		310	03-Feb-2014	Son of ID300. Unclear whether he also travelled.
10	Guangdong	284	27-Jan-2014	Father of ID289
		289	31-Jan-2014	Daughter of ID284
11	Guangdong	274	24-Jan-2014	Probable cluster (father)
		279	29-Jan-2014	Probable cluster (child, cousin of ID293)
		293	26-Jan-2014	Probable cluster (child, cousin of ID279)
12	Zhejiang	141	20-Nov-2013	Father in law of ID144
		144	29-Nov-2013	Son in law of ID141
13	Shandong	446	06-May-2014	Father of ID447
		447	15-May-2014	Son of ID446

*Data from news sources (CIDRAP, Recombinomics, Xinhua Net, South China Morning Post) and journal papers [9, 10]. Case ID refer to the linelist IDs.

Technical Appendix Table 3. Comparison of different market hazard functions in the absence of human-to-human transmission

Outbreak	Model	Likelihood	Parameters	BIC
Guangdong (2nd wave)	3 step	-150.0	5	326.7
	4 step	-146.1	7	329.5
	5 step	-141.9	9	331.8
	6 step	-139.6	11	337.7
	7 step	-140.2	13	349.6
Zhejiang (2nd wave)	3 step	-101.6	5	229.8
	4 step	-98.0	7	233.3
	5 step	-97.8	9	243.5
	6 step	-95.4	11	249.3
	7 step	-96.3	13	261.7

Technical Appendix Table 4. Estimated change in R_0 between 2014 and 2013 for influenza A/H7N9 outbreaks in Jiangsu and Zhejiang provinces*

Outbreak	Serial interval	$R_0^{2014} – R_0^{2013}$ (95%CI)	p-value
Jiangsu	3	-0.14(-0.73-0.48)	0.606
	5	-0.08(-0.56-0.50)	0.721
	7	-0.10(-0.61-0.23)	0.581
	9	-0.01(-0.45-0.48)	0.957
Zhejiang	3	0.25(-0.50-0.72)	0.448
	5	0.27(-0.16-0.62)	0.181
	7	0.28(0.01-0.61)	0.045
	9	0.20(0.04-0.44)	0.020

*Two-sided p-values are given for null hypothesis that R_0 is the same in both outbreak waves.

Technical Appendix Table 5. Estimated difference in market hazard reduction between Guangdong and other geographic regions*

Region	Outbreak wave	Serial interval	Difference in hazard reduction	p-value
Shanghai	1st	3	0.28(0.10-0.59)	0.006
		5	0.26(0.09-0.49)	0.003
		7	0.26(0.09-0.46)	0.003
		9	0.25(0.09-0.45)	0.002
Jiangsu	1st	3	0.25(-0.02-0.56)	0.059
		5	0.23(-0.02-0.46)	0.061
		7	0.24(0.01-0.45)	0.044
		9	0.23(-0.01-0.43)	0.053
Zhejiang	1st	3	0.29(0.12-0.59)	0.001
		5	0.27(0.10-0.49)	0.002
		7	0.26(0.09-0.46)	0.002
		9	0.26(0.09-0.45)	0.001
	2nd	3	0.27(0.09-0.58)	0.007
		5	0.25(0.08-0.47)	0.005
		7	0.24(0.07-0.45)	0.008
		9	0.23(0.07-0.43)	0.006

*Two-sided p-values are given for null hypothesis that there is no difference in hazard between Guangdong and specified region.

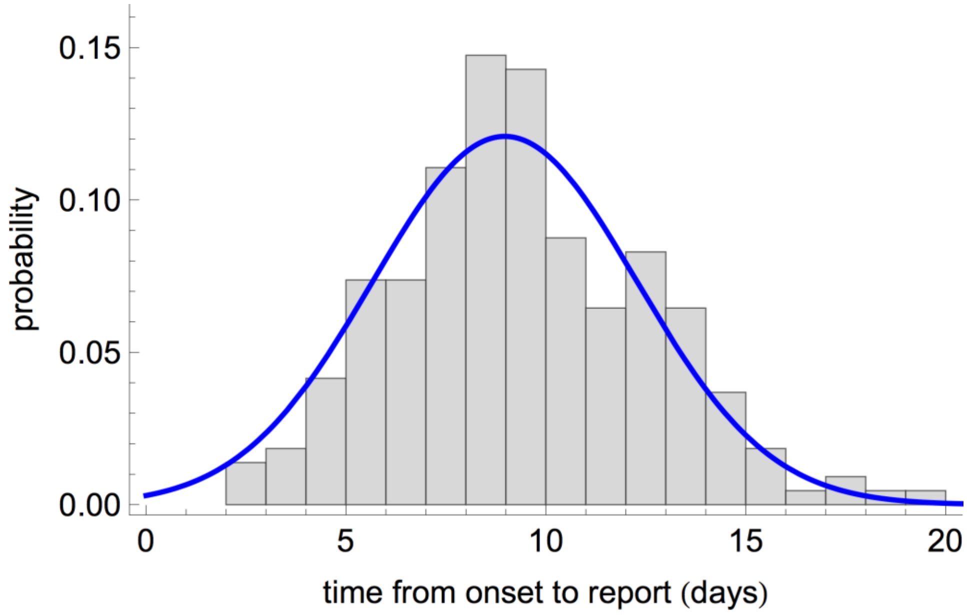

Technical Appendix Figure 1. Distribution of delay between case onset and report. We fitted a normal distribution (blue line) to influenza A/H7N9 cases reported between 19th February 2013 and 17th April 2014 (grey bars).

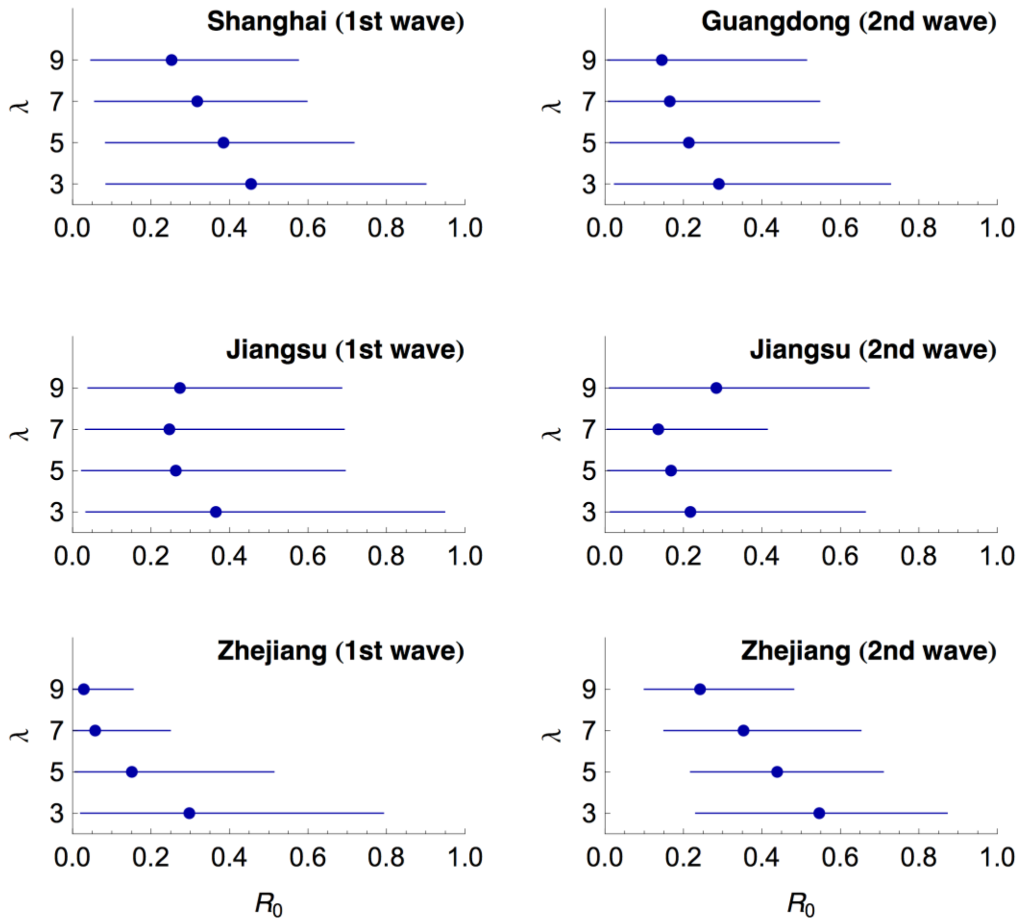

Technical Appendix Figure 2. Estimates of basic reproduction number in different regions as serial interval, *λ*, varies. Blue point, median of posterior estimate; blue line, 95% credible interval.

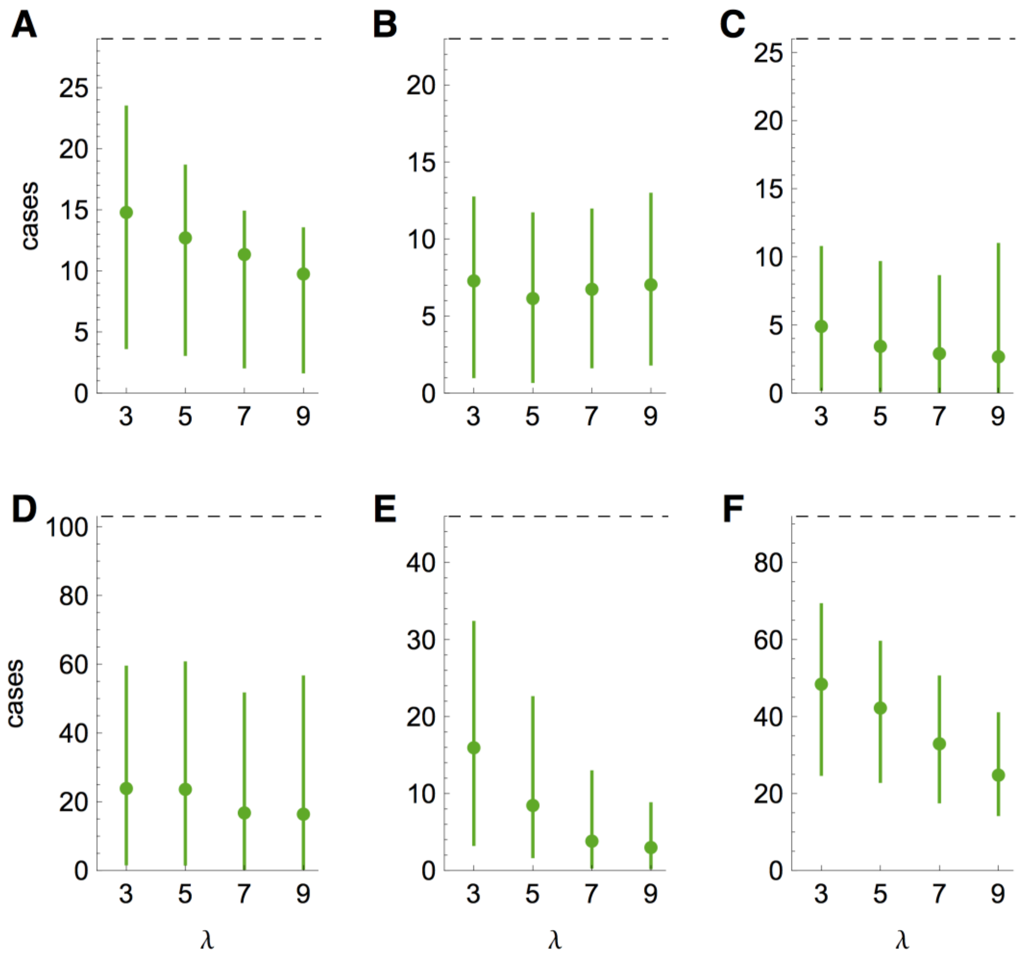

Technical Appendix Figure 3. Estimated human-to-human cases in different regions as serial interval, λ, varies. Dashed line, total reported cases; green point, estimated non-index cases; green line, 95% credible interval. (A) Shanghai (1st outbreak wave), (B) Jiangsu (1st wave), (C) Jiangsu (2nd wave), (D) Guangdong (2nd wave), (E) Zhejiang (1st wave), (F) Zhejiang (2nd wave).

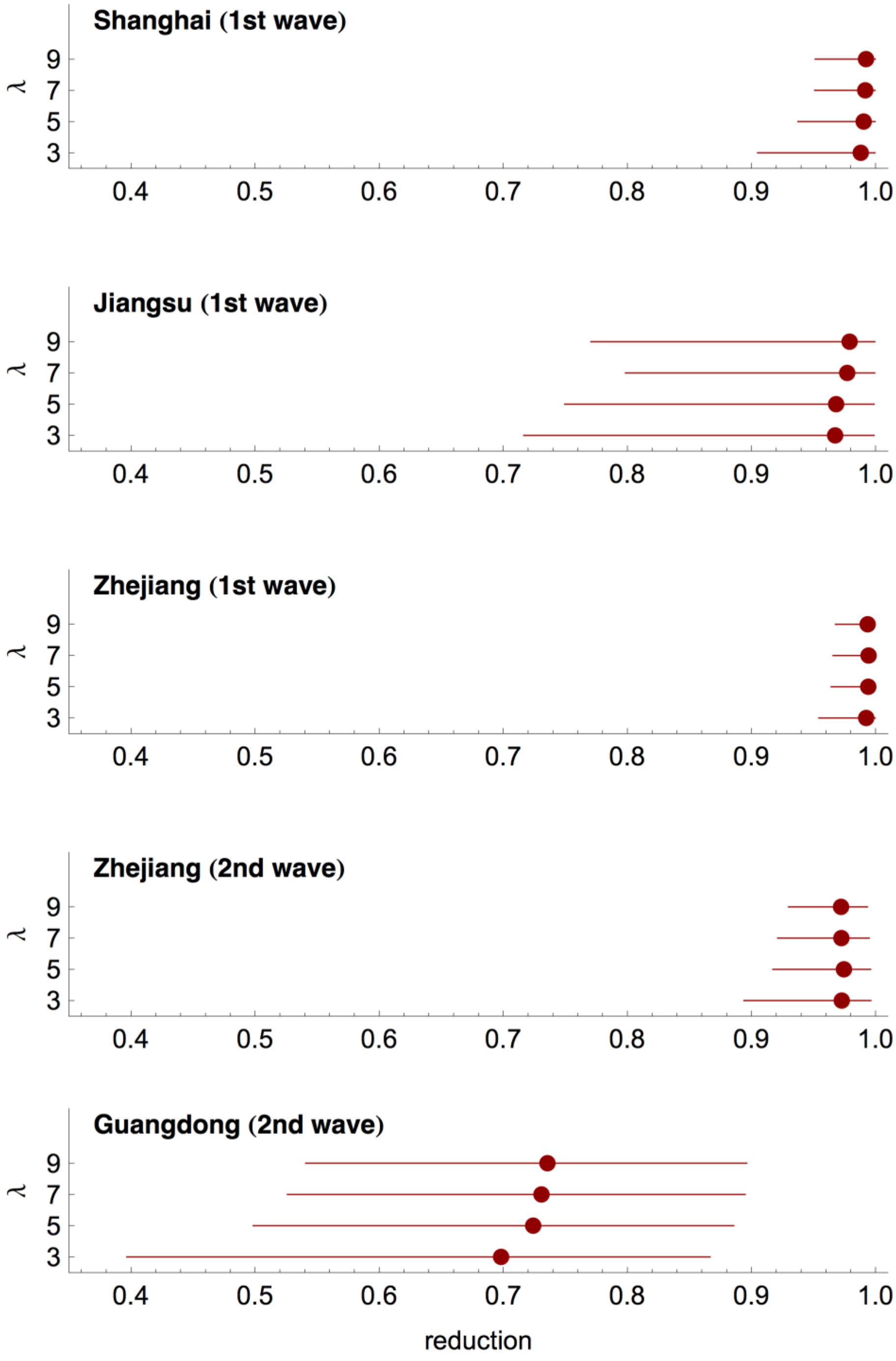

Technical Appendix Figure 4. Estimates of reduction in market hazard in different regions as serial interval, λ, varies.
